# Comparison of Text and Video Computer-Tailored Interventions for Smoking Cessation: Randomized Controlled Trial

**DOI:** 10.2196/jmir.3016

**Published:** 2014-03-03

**Authors:** Nicola Stanczyk, Catherine Bolman, Mathieu van Adrichem, Math Candel, Jean Muris, Hein de Vries

**Affiliations:** ^1^School for Public Health and Primary Care (CAPHRI)Department of Health PromotionMaastricht UniversityMaastrichtNetherlands; ^2^Faculty of Psychology and Educational SciencesOpen University of the NetherlandsHeerlenNetherlands; ^3^School for Public Health and Primary Care (CAPHRI)Department of Methodology and StatisticsMaastricht UniversityMaastrichtNetherlands; ^4^School for Public Health and Primary Care (CAPHRI)Department of Family MedicineMaastricht UniversityMaastrichtNetherlands

**Keywords:** smoking cessation, multiple computer tailoring, delivery strategy, educational level, text messages, video messages

## Abstract

**Background:**

A wide range of effective smoking cessation interventions have been developed to help smokers to quit. Smoking rates remain high, especially among people with a lower level of education. Multiple tailoring adapted to the individual’s readiness to quit and the use of visual messaging may increase smoking cessation.

**Objective:**

The results of video and text computer tailoring were compared with the results of a control condition. Main effects and differential effects for subgroups with different educational levels and different levels of readiness to quit were assessed.

**Methods:**

During a blind randomized controlled trial, smokers willing to quit within 6 months were assigned to a video computer tailoring group with video messages (n=670), a text computer tailoring group with text messages (n=708), or to a control condition with short generic text advice (n=721). After 6 months, effects on 7-day point prevalence abstinence and prolonged abstinence were assessed using logistic regression analyses. Analyses were conducted in 2 samples: (1) respondents (as randomly assigned) who filled in the baseline questionnaire and completed the first session of the program, and (2) a subsample of sample 1, excluding respondents who did not adhere to at least one further intervention session. In primary analyses, we used a negative scenario in which respondents lost to follow-up were classified as smokers. Complete case analysis and multiple imputation analyses were considered as secondary analyses.

**Results:**

In sample 1, the negative scenario analyses revealed that video computer tailoring was more effective in increasing 7-day point prevalence abstinence than the control condition (OR 1.45, 95% CI 1.09-1.94, *P*=.01). Video computer tailoring also resulted in significantly higher prolonged abstinence rates than controls among smokers with a low (ready to quit within 4-6 months) readiness to quit (OR 5.13, 95% CI 1.76-14.92, *P*=.003). Analyses of sample 2 showed similar results, although text computer tailoring was also more effective than control in realizing 7-day point prevalence abstinence. No differential effects were found for level of education. Complete case analyses and multiple imputation yielded similar results.

**Conclusions:**

In all analyses, video computer tailoring was effective in realizing smoking cessation. Furthermore, video computer tailoring was especially successful for smokers with a low readiness to quit smoking. Text computer tailoring was only effective for sample 2. Results suggest that video-based messages with personalized feedback adapted to the smoker’s motivation to quit might be effective in increasing abstinence rates for smokers with diverse educational levels.

**Trial Registration:**

Netherlands Trial Register: NTR3102; http://www.trialregister.nl/trialreg/admin/rctview.asp?TC=3102 (Archived by WebCite at http://www.webcitation.org/6NS8xhzUV).

## Introduction

A wide range of different smoking cessation interventions have been developed and implemented. In spite of this, smoking rates remain high, especially among people with lower levels of education [1-4]. This illustrates the need to improve smoking cessation intervention strategies for this group. Computer-tailored smoking cessation interventions have already shown to be effective in increasing abstinence rates [5-7]. A main characteristic of computer-tailored smoking cessation interventions is that respondents are provided with personalized feedback on their smoking behavior and motivational characteristics, such as attitudes, social support, self-efficacy, intentions, and action planning [8]. Compared to nontailored information, tailored messages enhance the processing of the health information and are more likely to be read, remembered, and perceived as personally relevant. [9-11]. Additionally, past research has indicated that the effects of tailoring can be enhanced by providing multiple tailored feedback moments [7,12] and has suggested a dose-response relationship between the number of feedback moments and smoking abstinence [13].

The Internet has become a promising method of delivering smoking cessation interventions and has increased opportunities to reach large numbers of people [14,15]. Although Web-based computer-tailored smoking cessation interventions have been shown to be potentially effective [15,16], they often report problems in attracting, engaging, and retaining smokers and quitters [7,17-20]. Smokers with a lower levels of education often leave the program before completing all intervention elements and show a lower adherence toward these programs [21-23]. Because smokers with lower levels of education appear to be more addicted, show fewer quit attempts, and are often more vulnerable to relapse [4,18,24], they constitute an important target group for participation in these computer-tailored smoking cessation interventions.

To date, Web-based computer-tailored smoking cessation interventions delivered via the Internet often consist of simple text-based messages. However, this might be not attractive enough for Internet users, especially less-educated groups [25]. Internet users often scan a text for relevant information but do not read the whole text [26,27]. Websites increasingly make use of pictures, graphics, and videos, and are often interactive to increase attractiveness [28]. Additionally, previous studies have suggested that the use of rich media, such as videos, may improve the appeal of health interventions [29-32] and may attract and stimulate comprehension among low health literacy groups [25,33,34]. Because video-based information seems to require less mental effort and may help the person to concentrate on the core elements of the message [35], the use of videos might be a possible strategy to attract, engage, and retain less-educated respondents in Web-based computer-tailored smoking cessation interventions [25]. In contrast, people with higher levels of education might profit more from in-depth processing; therefore, they may be more attracted by text-based messages (Soetens, K, personal communication, 2013). Studies have already tested the effects of a combination of interactive components, such as graphics, audio clips, and video clips [27,36,37], but to our knowledge no previous study has assessed the specific effect of tailored video-based messages on behavioral change and, in particular, on smoking cessation among groups with different levels of education.

Another strategy to improve the success of computer-tailored smoking cessation interventions is by focusing on the smoker’s motivation to quit smoking. Until now, most computer-tailored smoking cessation interventions have been developed for smokers with high motivation to quit [14,38], whereas less-educated smokers often show lower motivation to quit and might benefit from interventions which give them the possibility and time to reflect on their smoking behavior and intention to quit and to prepare successfully for their quit attempt. Consequently, Web-based computer-tailored smoking cessation interventions should be adapted to the needs of groups with different levels of education and should take the user’s motivation to quit into account.

The study described in this paper was designed to investigate the effectiveness of 2 computer-tailored smoking cessation interventions after 6 months: (1) a text-based multiple computer-tailored intervention where smokers received tailored text-based messages during several feedback moments, and (2) a video-based multiple computer-tailored intervention where smokers received tailored video-based messages during several feedback moments. In both interventions, smokers with high or low readiness to quit were able to choose different routings and received tailored feedback adapted to their readiness to quit. The effectiveness of the 2 interventions was compared to a control condition (respondents received a generic short text advice).

We hypothesized video-based computer tailoring to be more effective for smokers with a lower level of education, whereas text-based computer tailoring was expected to be more effective in smokers with a higher level of education. Because the interventions included different routings tailored according to the smokers’ readiness to quit, we expected less-motivated smokers to be equally successful in their quit attempts as more motivated smokers. Therefore, we explored whether the effects of the 2 interventions were different for individuals with a high or low readiness to quit. Moreover, we conducted our analyses in 2 different samples: (1) respondents who filled in baseline questionnaire and completed the first session of the program, and (2) a subsample of sample 1, excluding respondents who did not adhere to at least one further intervention session [7,13]. Finally, an overview of the program evaluation of respondents will be shown.

## Methods

### Ethics Approval and Registration

The current study was submitted for approval to the Medical Research Ethics Committee (MREC) of Atrium Medical Centre Heerlen. The MREC decided that no MREC approval was necessary because respondents were not required to undertake any particular action. The study was registered at the Dutch Trial Register (NTR3102). The study was in-line with the ethical codes of conduct of the American Psychological Association (APA) [39].

### Respondents and Recruitment

Respondents were recruited from December 2010 to June 2012 to participate in the Web-based multiple computer-tailored smoking cessation intervention. Respondents were eligible for participation if they were motivated to quit smoking within the next 6 months, were 18 years or older, and had access to the Internet.

Respondents were recruited by several channels. First, a random sample of approximately 150 general practitioners (GPs) was asked to refer smoking patients to the intervention website. The GP practices were provided with recruitment materials (flyers, business cards, etc) for this purpose. Second, respondents were also recruited to participate through advertising campaigns in local newspapers, newspaper websites, and Dutch health fund websites. Lastly, we used several national and international online social networking websites, such as Hyves and Facebook, to invite smokers to participate in our smoking cessation study. All advertisements provided a link to the intervention website that enabled people to find out more information about the intervention and participation.

### Design and Procedure

The current study was a randomized controlled trial with 2 experimental conditions (text-based computer tailoring vs video-based computer tailoring) and a control condition in which respondents received only a single generic short text advice. Interested respondents could sign up via the intervention website [40]. On the intervention website, respondents were informed that they could be randomly allocated to 1 of the 3 conditions and that they would have the chance to win €100 if they completed all the assessments (before decision to participate, registration, and baseline measurement). After creating a personal log-in and account, respondents were randomized into 1 of the 3 conditions. Respondents were not told about the content of the other experimental condition.

**Figure 1:**
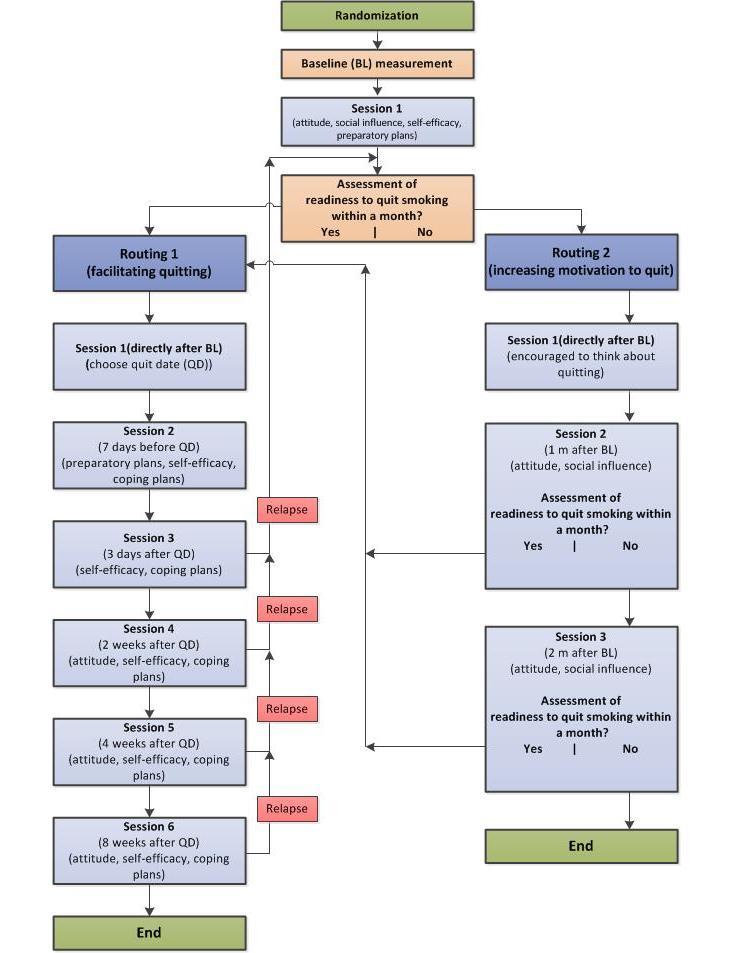
Intervention design of a video- and text-based computer-tailored intervention for smoking cessation following 2 routings.

After giving online informed consent, respondents were asked to fill out the baseline questionnaire. Respondents in the text-based and video-based condition received tailored feedback over 3 months (see Intervention and Figure 1 for details). At 6-month follow-up, all respondents were sent an email invitation with a link to the intervention website to fill out the 6-month follow-up measurement. Respondents who did not complete the follow-up measurement after 1 week were reminded by email to fill out the online questionnaire. A further reminder was sent after 2 weeks if necessary. Respondents who did not respond to the email invitation or the 2 reminders received another email, inviting them to briefly indicate their current smoking status. This email requested completion of a shortened version of the 6-month follow-up measurement, consisting of 10 (instead of 95) important smoking-related questions, which they could return by email. Lastly, if this abbreviated email assessment was still not completed, respondents were called for a short telephone interview, asking the same questions as in the shortened online questionnaire.

### Intervention

The 2 Web-based multiple computer-tailored smoking cessation interventions (text-based vs video-based computer tailoring) varied only in their mode of delivery (see Figure 1). The intervention was based on 2 previously tested computer-tailored interventions which were found to be effective in smoking cessation [6,12]. The I-Change model, integrating various social cognitive theories [41-43], was used as a theoretical framework of the currently tested intervention. After completing the baseline assessment, respondents in the 2 experimental conditions first received tailored feedback on their smoking behavior, followed by feedback about their attitude (pros and cons of smoking and quitting), their perceived social influence (modeling and support), their perceived self-efficacy, and how to prepare to quit (eg, how to plan a quit date). Next, respondents were asked whether they wanted to quit within a month. Depending on this readiness to quit smoking within the following month, respondents received more personalized feedback during subsequent multiple computer-tailored sessions, defined in routing 1 or 2. These routings varied between those who already wanted to set a quit date within a month compared to those who did not plan a quit date in the forthcoming month (see Figure 1). Respondents in the 2 experimental conditions (text-based vs video-based computer tailoring) also received an overview of the tailored advice by email after each session.

### Routing 1

Respondents who had set a goal to quit within 1 month were directed to routing 1.The goal of routing 1 was to help smokers translate their intention to quit into action by providing tailored feedback to increase self-efficacy and effective action planning. In the first session, after receiving feedback on their smoking behavior, attitude, social influences, and self-efficacy with respect to quitting, respondents were asked to choose a quit date (between 8 days and 1 month from the first session). At the end of this first session, respondents were informed that they would be invited to the next session 1 week before their quit date, to receive help with quitting. During the second session (1 week before their quit attempt), respondents received feedback on the extent to which they had already made concrete plans for their quit attempt because past research has revealed that preparing for quitting increases the likelihood of quitting [6]. In addition, they received feedback on their perceived self-efficacy, including tailored feedback on coping planning to help them to deal with difficult situations that may cause relapse. Different studies have found that ex-smokers often relapse shortly after their quit attempt; therefore, respondents were provided with different relapse prevention strategies as described previously [42,44,45]. During the third session, 3 days after their quit attempt, feedback was given on the quitter’s perceived self-efficacy. Respondents also received personalized tips on how to deal with personal risk situations and were invited to formulate coping plans again to prevent potential relapse. During the fourth session, 2 weeks after their quit date, respondents received tailored feedback on their perceived self-efficacy, including feedback on how to deal with difficult situations and attitudes toward smoking and quitting (perceived pros and cons of smoking and quitting). In sessions 5 and 6 at 4 and 8 weeks, respectively, after their quit date, a similar strategy was used as for session 4. Respondents could choose to receive feedback on different items (eg, how to cope with negative moods with coping plans or self-efficacy items about how to deal with difficult risk situations). This option was provided because we expected respondents to encounter different problems throughout their quit attempt. During all sessions, respondents were invited to continue their quit attempt or, if they had relapsed, to indicate their readiness to quit smoking and plan a new quit date. Respondents could restart their quit attempt several times (no maximum) during the program, if they wanted.

### Routing 2

Respondents who were not ready to quit within 1 month were directed to routing 2. The goal of routing 2 was to increase motivation by increasing perception of the pros of quitting and knowledge of how to obtain support for quitting. In session 1, directly after completion of the baseline assessment, smokers were encouraged to use the following month to reflect on their smoking behavior and motivation to quit. In session 2, 1 month after baseline, respondents were invited by email for the next session. Respondents received tailored feedback on their smoking behavior, their attitude (pros and cons of smoking and quitting), and their perceived social support. Next, they were invited to indicate their readiness to quit smoking. Respondents who indicated an intention to quit within 1 month were directed to routing 1 and were asked to set a quit date. Respondents who were not ready to quit received an invitation to take part in the next session (session 3); this session used a similar strategy that was used in session 2. Respondents ready to quit were directed to routing 1 and were asked to set a quit date. Respondents who indicated at the end of session 3 that they were not prepared to quit received a kind message indicating that the intervention program would respect the fact that they were not ready to quit smoking and that they would receive no further invitations.

### Mode of Delivery

The content of the feedback messages was exactly the same in both the text- and video-based conditions. In the text-based condition, respondents received multiple sessions of text-based computer-tailored advice without any graphics or animations. In the video-based condition, the same tailored advice was presented by adults in a video message. Five different adult presenters (2 males, 3 females) were selected out of a screening test of 20 persons who delivered the tailored advice in a TV news program format. We used a mix of adults during the different sessions who presented the different pieces of tailored advice.

### Measurements

#### Baseline Measurement

The following demographic variables were assessed: age, gender (0=male; 1=female), educational level (1=low corresponding to primary, basic vocational, lower general school, or no education; 2=intermediate corresponding to higher general secondary education, preparatory academic education, or medium vocational school; 3=high corresponding to higher vocational school or university level), and nationality (0=other nationality; 1=Dutch nationality).

Addiction level was measured by 6 items using the Fagerström Test for Nicotine Dependence (FTND), asking respondents how many cigarettes they smoked per day, at which time points, and whether they had difficulties not smoking in smoke-free places (0=not addicted; 10=highly addicted) [46].

Readiness to quit smoking was assessed with a single item asking respondents whether and when they intended to quit smoking, resulting in 3 categories (1=yes, within 4 to 6 months; 2=yes, within 1 to 3 months; 3=yes, within the following month) [43].

Smoking habit was assessed using an abbreviated version of Verplanken and Orbell’s Self-Reported Habit Index of 6 items (eg, smoking is something which I do automatically) with which respondents could agree or disagree, resulting in a 5-point scale (1=I totally disagree; 5=I totally agree). A mean scale score was included in the analyses (Cronbach alpha=.78) [47].

Depressive symptoms were measured with the abbreviated 10-item Center for Epidemiologic Studies Depression scale (CES-D) that asked respondents whether they felt depressed during the past week, for example, resulting in a 4-point scale (1=rarely or none of the time; 4=most or all of the time) [48]. A sum score was included in the analyses (Cronbach alpha=.85).

Occurrence of smoking-related diseases was measured by 4 questions on a dichotomous scale, such as “Do you suffer from chronic obstructive pulmonary disease (COPD), cancer, diabetes, or cardiovascular disease?” (0=no; 1=yes).

Attitude was measured by 3 items assessing the pros and cons of quitting (quitting smoking would be reasonable, bad, or enjoyable), resulting in a 5-point scale (1=I totally disagree; 5=I totally agree). A mean scale score was included in the analyses (Cronbach alpha=.52). A higher score represents a positive attitude toward quitting.

Social influence was measured by 2 scales: a social modeling and a social support scale. Social modeling was assessed by 2 items that measured whether other people in their environment smoked, such as partners (1=no, 2=yes, 9=not applicable), and in their social environment, such as family or friends (1=none, 2=a minority, 3=half, 4=a majority, 5=all, 9=not applicable). A total of 552 respondents for the partner question and 80 respondents for the social environment question filled in “not applicable” when they were asked whether their partner or their social environment smoked. Social support was measured with 2 items that asked whether smokers received social support (partners and social environment, respectively) in favor of quitting on a 4-point scale (1=no, 2=yes, a bit, 3=yes, moderate, 4=yes, a lot, 9=not applicable). A total of 787 respondents for the partner question and 229 for the social environment question filled in “not applicable” when they were asked whether they received support from their partner or their social environment. Not applicable was recoded into the lowest value (1=no support) for the social influence measure. The items were summed and formed an index that was included in the analyses.

Preparatory plans were assessed by 3 items that measured whether participants planned to execute different preparatory plans for their quit attempt (removing ashtrays, telling their environment to quit smoking, quitting without decreasing smoking first) on a 5-point scale (1=surely not; 5=surely yes). The items were summed and formed an index that was included in the analyses.

Coping plans were assessed by 4 items that measured whether participants had made specific plans to prevent relapse in difficult situations, such as plans how to cope with negative mood, plans how to cope when being at a party or drinking a cup of coffee, or being offered a cigarette (0=no; 1=yes). Difficult situations were selected and predefined based on previous studies [6,12,45]. The items were summed and formed an index that was included in the analyses.

Self-efficacy was measured by 3 items asking respondents whether they would be able to refrain from smoking in these difficult situations (Do you think you will manage not to smoke when you drink a cup of coffee, when you are in a negative mood, or when you visit a party?), resulting in a 5-point scale (1=definitely not; 5=yes, definitely). A mean scale score was included in the analyses (Cronbach alpha=.62).

The variables attitude, self-efficacy, preparatory plans, and coping plans were also used to determine the tailored advice during the first session of the intervention.

#### Follow-Up Measures

At the 6-month follow-up measurement, 7-day point prevalence abstinence was self-assessed by 1 item asking respondents whether they had refrained from smoking during the past 7 days (0=no; 1=yes) [49,50].

In addition, prolonged abstinence was self-assessed by 1 item asking respondents whether they had refrained from smoking since their last quit attempt allowing for a 2-week grace period during which the respondent could smoke 1 to 5 cigarettes (0=no;1=yes) [49,50]. In-line with the definition of prolonged abstinence, those who reported that they had quit less than 3 months before the follow-up measurements were not included as quitters in the prolonged abstinence measurement [50].

#### Evaluation of the Program

Process evaluation was conducted by measuring 5 concepts, each measured on a 5-point scale (1=totally disagree to 5=totally agree):

1. Attention to the tailored advice (eg, the advice was interesting) was measured by 3 items (Cronbach alpha=.94).
2. Comprehension of the advice (eg, the advice was clear to me) was measured by 3 items (Cronbach alpha=.78).
3. Adaptation toward the advice (eg, the advice was personally relevant for me) was measured by 3 items (Cronbach alpha=.79).
4. Appreciation of the advice (eg, I appreciated the advice) was measured by 3 items (Cronbach alpha=.93).
5. Processing of the advice (eg, the advice encouraged me to think more about smoking cessation) was measured by 8 items. For all process evaluation scales a mean scale score was included in the analyses (Cronbach alpha=.91).

### Statistical Analysis

The inclusion of all randomly assigned respondents is a common approach to analyze the effects of an intervention [51,52]. Because not all respondents (video and text conditions) adhered to all intervention elements, the inclusion of these respondents in the effect analyses might distort the assessment of an intervention’s effectiveness. It might be adequate to include only respondents in the analyses who actually followed the intervention for at least one session [7]. Therefore, we chose to analyze 2 different samples. The first sample included all randomly assigned respondents that filled in baseline questionnaire and session 1 (directly after baseline assessment, including setting a quit date). The second sample included only respondents in the experimental conditions who finished at least one further session of the 2 different routings of the intervention.

As a preliminary, descriptive analyses were conducted to check for baseline differences between the 3 conditions. Chi-square tests were used for categorical variables whereas analyses of variance (ANOVAs) were used for continuous variables. If the chi-square test showed a *P* value <.05, post hoc pairwise comparisons with Bonferroni correction (alpha=.05/3=.017) were used. If the overall *F* test showed a *P* value <.05, the Tukey honestly significant difference (HSD) method was used for post hoc pairwise comparisons. Second, logistic regression was used to analyze attrition, including baseline factors and condition as predictors. Baseline differences and significant predictors of dropout were included in all logistic regression effect analyses explained subsequently.

Third, logistic regression analyses were conducted to investigate the effectiveness of the intervention on the outcome measures assessed at the 6-month follow-up measurements. The analyses were performed adjusting for potential confounders, including demographic variables (eg, age, educational level, gender, and ethnicity) and possible moderators of the intervention effect (eg, addiction level, recruitment strategy, readiness to quit smoking, depression, smoking-related illnesses, self-efficacy, preparatory planning, and coping planning), baseline differences, dropout predictors and 2 interaction terms (readiness to quit smoking by condition and educational level by condition). Where significant interaction terms were found, stratified analyses were performed separately for each group.

In the effect analyses, a negative scenario was used in which every respondent missing at follow-up was regarded as a smoker. In addition, we also used multiple imputation [53] to fill in missing values. Missing values for outcome variables were imputed based on the regression of all relevant variables that were used in the main effect analyses. The number of imputations was set at 30. This was done according to the recommendation to create as many imputed datasets as the percentage of cases with missing data [54].

Lastly, we also conducted complete case analyses, in which we only took respondents into account who filled out the 6-month follow-up measurements (these results are presented in Multimedia Appendix 1). Data were analyzed using SPSS 19.0 (SPSS, Inc, Chicago, IL, USA).

## Results

### Sample Characteristics and Attrition Analysis

**Figure 2:**
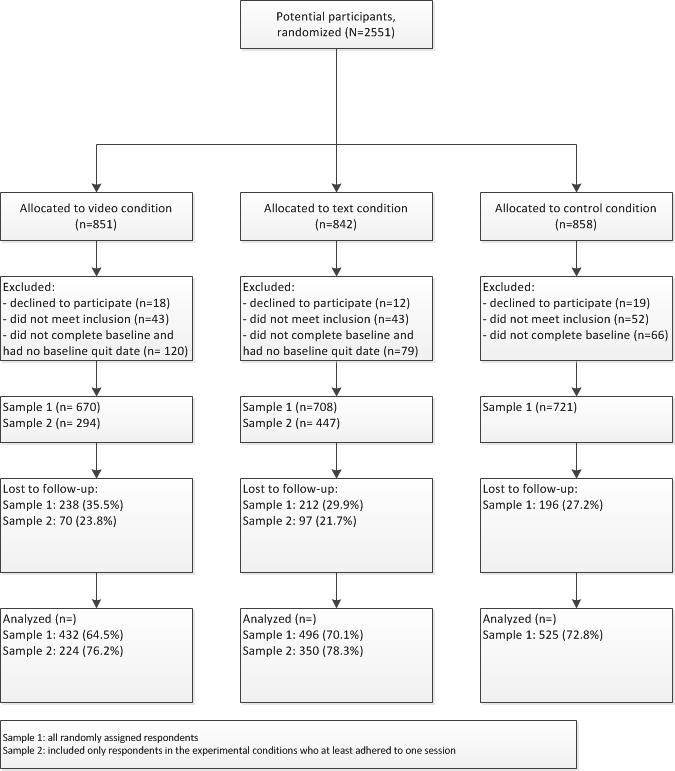
Flowchart of participant enrollment and inclusion. Sample 1: all randomly assigned respondents; sample 2: only respondents in the experimental conditions who adhered to at least one session.

Figure 2 shows the flow of respondents for the 3 conditions. Of the 2551 potential respondents who were randomized to 1 of the 3 conditions, 49 (1.92%) declined to participate, 138 (5.41%) did not meet inclusion criteria, and 265 (10.39%) did not complete the baseline questionnaire or had no baseline quit date (within routing 1). As Figure 2 illustrates, eligibility was checked after randomization. The different tailored feedback sessions were organized around the quit date; therefore, respondents had to fill in their quit date (in the experimental conditions) otherwise they were excluded from the study. Finally, 2099 respondents were included in the video-based computer tailoring condition (n=670), the text-based computer tailoring condition (n=708), and the control condition (n=721).

Table 1 shows the characteristics of the total sample and the baseline differences between the 3 conditions in terms of demographic and smoking-related variables. Mean age of respondents was 45.7 years (SD 12.8). Of the 2099 respondents, 1278 (60.88%) were female and 705 (33.58%) had a low level of education. Furthermore respondents smoked on average approximately 19 (SD 8.6) cigarettes per day. Respondents in the 3 conditions differed significantly in terms of readiness to quit smoking. Respondents in the video-based and text-based computer tailoring conditions were less ready to quit smoking than respondents in the control condition. Moreover, there were also differences between the 3 conditions regarding preparatory planning and coping planning. Respondents in the 2 experimental conditions were more likely to have made preparatory and coping plans compared to respondents in the control condition. In sample 1, 238 of 670 (35.5%) were lost to follow-up in the video-based computer tailoring condition, versus 212 of 708 (29.9%) in the text-based computer tailoring condition and 196 of 721 (27.2%) in the control condition. Attrition analysis showed that respondents were significantly more likely to complete the follow-up assessment if they were in the text-based computer tailoring and control condition (OR 1.32, 95% CI 1.05-1.67, *P*=.02; OR 1.47, 95% CI 1.16-1.87, *P*=.001, respectively), if recruited by Internet or newspaper advertisements (OR 0.62, 95% CI 0.40-0.97, *P*=.04), if they were older (OR 1.02, 95% CI 1.01-1.03, *P*=.001), were of Dutch nationality (OR 1.63, 95% CI 1.06-2.52, *P*=.03), and had higher levels of self-efficacy (OR 1.12, 95% CI 1.06-1.33, *P*=.003). Baseline differences and significant predictors at dropout were included in all further analyses as potential confounders.

**Table 1:** Baseline sample characteristics for the video-based computer tailoring (video), text-based computer tailoring (text), and control conditions (recruited between December 2010 and June 2012).

Variables		Overall sample (N=2099)	Video (n=670)	Text (n=708)	Control (n=721)	*P* ^a^	Tukey HSD/ Bonferroni^b^
Gender (female), n (%)		1278 (60.9)	417 (62.2)	431 (60.9)	430 (59.6)	.61	
Age (years), mean (SD)		45.7 (12.8)	45.5 (13.0)	45.4 (12.8)	46.2 (12.5)	.46	
**Educational level, n (%)**						.41	
	Low	705 (33.6)	225 (33.6)	231 (32.6)	249 (34.5)		
	Medium	782 (37.3)	247 (36.9)	255 (36.0)	280 (38.8)		
	High	612 (29.2)	198 (29.5)	222 (31.4)	192 (26.6)		
Dutch nationality, n (%)		1995 (95.2)	639 (95.5)	674 (95.2)	682 (94.9)	.85	
FTND^c^ score (1-10), mean (SD)		4.9 (2.4)	5.0 (2.3)	4.9 (2.4)	4.9 (2.5)	.46	
Number of cigarettes smoked per day, mean (SD)		18.8 (8.6)	19.0 (8.1)	18.7 (8.4)	19.0 (9.2)	.75	
**Readiness to quit, n (%)**						.005	Video/text<control
	Within 1 month	1093 (52.1)	368 (54.9)	384 (54.2)	341 (47.3)		
	Within 1-3 months	636 (30.3)	205 (30.6)	203 (28.7)	228 (31.6)		
	Within 4-6 months	370 (17.6)	97 (14.5)	121 (17.1)	152 (21.1)		
**Diseases, n (%)**							
	With COPD diseases	290 (13.8)	97 (14.5)	99 (14.0)	94 (13.0)	.73	
	With cancer	34 (1.6)	10 (1.5)	9 (1.3)	15 (2.1)	.46	
	With diabetes	99 (4.7)	27 (4.0)	33 (4.7)	39 (5.4)	.48	
	With cardiovascular diseases	210 (10.0)	63 (9.3)	60 (8.5)	87 (12.1)	.06	
	With asthmatic diseases	171 (8.1)	63 (9.4)	57 (8.1)	51 (7.1)	.28	
**Recruitment strategy, n (%)**						.76	
	General practitioner	166 (7.9)	56 (8.4)	57 (8.1)	53 (7.4)		
	Newspaper/Internet	1631 (77.7)	511 (76.3)	551 (77.8)	569 (78.9)		
	Family/friends	203 (9.7)	69 (10.3)	72 (10.2)	62 (8.6)		
	Other strategies	99 (4.7)	34 (5.1)	28 (4.0)	37 (5.1)		
Depressive feelings		5.8 (2.4)	5.9 (2.5)	5.8 (2.4)	5.8 (2.4)	.42	
Habit		4.0 (0.6)	4.0 (0.6)	4.0 (0.6)	4.0 (0.7)	.50	
Social support		5.1 (1.8)	5.1 (1.7)	5.2 (1.8)	5.1 (1.9)	.41	
Social modeling		3.9 (1.2)	4.0 (1.2)	4.0 (1.3)	3.9 (1.2)	.15	
Attitude		4.2 (0.7)	4.2 (0.7)	4.1 (0.7)	4.2 (0.7)	.53	
Self-efficacy		3.2 (0.9)	3.2 (0.9)	3.1 (0.9)	3.1 (0.9)	.22	
Preparatory planning		11.0 (2.6)	11.2 (2.6)	11.0 (2.6)	10.8 (2.6)	.008	Video>control
Coping planning		1.2 (1.5)	1.5 (1.6)	1.3 (1.5)	0.96 (1.5)	<.001	Video/text>control

^a^Analyses of variance (ANOVAs, *F* test) were used for continuous variables; chi-square tests were used for categorical variables.

^b^Tukey honestly significant difference (HSD), alpha=.05; Bonferroni-corrected alpha=.05/3=.017.

^c^FTND: Fagerström Test for Nicotine Dependence.

### Quit Rates at Six-Month Follow-Up

Table 2 shows the raw abstinence rates for sample 1 for the negative scenario. When respondents lost to follow-up were regarded as smokers (negative scenario), 7-day point prevalence abstinence rates in sample 1 were 20.9% in the video-based computer tailoring condition, 17.9% in the text-based computer tailoring condition, and 14.6% in the control condition. In sample 2, 7-day point prevalence abstinence rates were 30.6% in the video-based computer tailoring condition, 22.6% in text-based computer tailoring condition, and 14.6% in the control condition (see Multimedia Appendix 1). Prolonged abstinence rates are also presented stratified by readiness to quit smoking. In sample 1, prolonged abstinence rates in the video-based computer tailoring, text-based computer tailoring, and the control condition (for people with a lower readiness to quit over 4 to 6 months) were 14.4%, 8.3%, and 3.3%, respectively. Finally, sample 2 showed prolonged abstinence rates (for people with a lower readiness to quit within 4 to 6 months) of 23.1%, 9.5%, and 3.3%, respectively, in the video-based computer tailoring, text-based computer tailoring, and the control conditions (see Multimedia Appendix 1).

**Table 2:** Six-month abstinence rates, including 7-day point prevalence abstinence (PPA) and prolonged abstinence (PA), for the video-based computer tailoring (video), text-based computer tailoring (text), and control conditions for sample 1 (negative scenario).

Negative scenario		Total, N	Condition, n (%)			*P* value
			Video	Text	Control	
**7-Day PPA**		2099	140 (20.9)	127 (17.9)	105 (14.6)	.008
	PA	2099	98 (14.6)	99 (14.0)	87 (12.1)	.34
	PA	2099				
**Readiness to quit**						
	Within 1 month		62 (16.8)	71 (18.5)	52 (15.2)	.51
	Within 1-3 months		22 (10.7)	18 (8.9)	30 (13.2)	.36
	Within 4-6 months		14 (14.4)	10 (8.3)	5 (3.3)	.006

### Differences in Point Prevalence Abstinence Between Conditions at Follow-Up

When respondents lost to follow-up were regarded as smokers in the analyses, no significant interaction was found between the type of condition and educational level (χ^2^_4_=6.3, *P*=.18) nor between condition and respondents’ readiness to quit smoking (χ^2^_4_=3.1, *P*=.54) on 7-day point prevalence abstinence. Our analysis, however, revealed a main intervention effect on 7-day point prevalence abstinence. In sample 1 (including all respondents as randomly assigned), video-based computer tailoring was significantly more effective than the control condition (OR 1.45, 95% CI 1.09-1.94, *P*=.01). In sample 2 (with only those who followed at least one further session of the intervention), both experimental conditions were significantly more effective than the control condition (video-based computer tailoring: OR 2.29, 95% CI 1.64-3.20, *P*<.001; text-based computer tailoring: OR 1.57, 95% CI 1.15-2.15, *P*=.005) (see Multimedia Appendix 1). In sample 2, the video-based computer tailoring condition was significantly more effective than the text-based computer tailoring condition (OR 1.46, 95% CI 1.04-2.05, *P*=.03) Other predictors of 7-day point prevalence abstinence were a higher readiness to quit, a lower degree of depressive symptoms, making more preparatory plans, having a higher self-efficacy, and being recruited by GPs (see Table 3). The multiple imputation procedure revealed similar results. In samples 1 and 2, video-based computer tailoring was significantly more effective than the control condition (sample 1: OR 1.55, 95% CI 1.16-2.08, *P*=.003; sample 2: OR 1.93, 95% CI 1.38-2.70, *P*<.001). The text-based computer tailoring condition in sample 2 did not reach significance when compared to the control condition (text-based computer tailoring: OR 1.31, 95% CI 0.96-1.78, *P*=.09). Complete case analyses revealed comparable results (see Multimedia Appendix 2).

**Table 3:** Factors associated with 7-day point prevalence abstinence in sample 1 (negative scenario) in the present study.

Negative scenario variables^a^	Sample 1 (N=2099)		
	OR	95% CI	*P* value
Video vs control	1.45	1.09-1.94	.01
Text vs control	1.22	0.92-1.63	.17
Gender (male)	0.90	0.70-1.14	.38
Age	1.01	1.00-1.02	.07
Dutch nationality	1.23	0.71-2.15	.46
Middle education level^b^	1.08	0.81-1.45	.58
High education level^b^	1.17	0.87-1.59	.30
Readiness to quit within 1 month^c^	1.71	1.16-2.50	.006
Readiness to quit within 1-3 months^c^	1.41	1.17-2.10	.09
FTND score	0.96	0.91-1.00	.07
CES-D score	0.94	0.89-.99	.03
With COPD^d^	1.03	0.71-1.50	.86
With cancer^d^	1.00	0.40-2.50	.99
With diabetes^d^	1.22	0.69-2.20	.51
With cardiovascular diseases^d^	1.18	0.78-1.78	.43
With asthma^d^	0.89	0.58-1.39	.61
Recruitment strategy, newspaper/Internet^e^	0.67	0.45-0.99	.04
Preparatory planning	1.07	1.02-1.12	.009
Coping planning	1.01	0.94-1.10	.72
Self-efficacy	1.15	1.01-1.33	.04

^a^Interaction terms are not included in the final model because they were not significant and ORs are adjusted for variables significant at baseline and dropout.

^b^Low education is the reference category.

^c^Willingness to quit within 4-6 months is the reference category.

^d^Not suffering from the disease is the reference category.

^e^General practitioner (GP) is the reference category.

### Differences in Prolonged Abstinence Between Conditions at Follow-up

In the negative scenario, no significant interaction was found between condition and educational level on prolonged abstinence (χ^2^_4_=3.1, *P*=.54). However, analysis revealed a significant interaction effect between the type of intervention and respondents’ readiness to stop smoking with regard to prolonged abstinence in sample 1 (χ^2^_4_=12.0, *P*=.02). Subsequent subgroup analysis showed that video-based computer tailoring was significantly more effective than the control condition in increasing prolonged abstinence rates among respondents who were less motivated to quit (ie, those who had stated they were ready to quit within 4 to 6 months; see subgroup analyses in Table 4). Similarly, in sample 2, a significant interaction was found between the type of intervention and respondents’ readiness to quit smoking with regard to prolonged abstinence (χ^2^_4_=10.6, *P*=.03). Subsequent subgroup analysis showed that video-based computer tailoring was more effective in increasing prolonged abstinence rates among respondents with high (within 1 month) and low (within 4-6 months) readiness to quit (see Multimedia Appendix 1). The multiple imputation analyses yielded similar results. A significant interaction was shown, in which the video-based computer tailoring condition was still effective for smokers who were ready to quit within 4 to 6 months in both samples (sample 1: OR 2.75, 95% CI 0.94-8.10, *P*=.06; sample 2: OR 3.17, 95% CI 0.94-10.73, *P*=.06). The complete case analyses yielded similar results compared with the other analyses (see Multimedia Appendix 2).

**Table 4:** Factors associated to prolonged abstinence in sample 1 (negative scenario) in the present study.

Negative scenario			Sample 1 (N=2099)		
			OR^a^	95% CI	*P* value
**Variable**					
	Video vs control		5.13	1.76-14.92	.003
	Text vs control		2.79	0.92-8.46	.07
	Gender (male)		0.72	0.54-0.95	.02
	Age		1.01	1.00-1.02	.05
	Dutch nationality		1.31	0.69-2.44	.40
	Middle education level^b^		1.16	0.84-1.61	.35
	High education level^b^		1.01	0.72-1.43	.94
	Readiness to quit within 1 month^c^		4.18	1.61-10.85	.003
	Readiness to quit within 1-3 months^c^		4.11	1.55-10.95	.005
	FTND score		0.95	0.89-1.00	.04
	CES-D score		0.90	0.84-.96	.002
	With COPD^d^		1.23	0.80-1.89	.34
	With cancer^d^		0.66	0.26-1.66	.37
	With diabetes^d^		1.04	0.55-1.96	.90
	With cardiovascular diseases^d^		1.24	0.78-1.89	.37
	With asthma		1.12	0.67-1.90	.66
	Recruitment strategy, newspaper/Internet^e^		0.62	0.41-0.95	.03
	Preparatory planning		1.08	1.02-1.14	.007
	Coping planning		1.07	0.98-1.17	.14
	Self-efficacy		1.18	1.01-1.38	.04
**Interactions**					
	High readiness to quit × video		0.21	0.07-0.66	.007
	High readiness to quit × text		0.45	0.12-1.45	.18
	Middle readiness to quit × video		0.15	0.04-0.51	.002
	Middle readiness to quit × text		0.23	0.06-0.81	.02
**Subgroup analyses**					
	**Readiness to quit within 1 month**				
		Video vs text	0.86	0.59-1.27	.46
		Video vs control	1.07	0.71-1.62	.74
		Text vs control	1.24	0.83-1.86	.29
	**Readiness to quit within 1-3 months**				
		Video vs text	1.23	0.63-2.40	.54
		Video vs control	0.77	0.43-1.41	.40
		Text vs control	0.63	0.34-1.18	.15
	**Readiness to quit 4-6 within months**				
		Video vs text	1.84	0.77-4.40	.17
		Video vs control	5.13	1.76-14.92	.003
		Text vs control	2.80	0.92-8.46	.07

^a^ORs are adjusted for variables significant at baseline and dropout.

^b^Low education is the reference category.

^c^Willingness to quit within 4-6 months is the reference category.

^d^Not suffering from the disease is the reference category.

^e^General practitioner (GP) is the reference category.

### Adherence to the Intervention

Table 5 presents the 7-day point prevalence abstinence rates for the 2 experimental conditions. Results showed significant higher abstinence rates for higher-educated smokers in the text condition. Table 5 also shows the 7-day point prevalence abstinence rates for those who did and who did not adhere to at least one further intervention element. Abstinence rates are presented separately for the 2 experimental conditions and are stratified by educational level. The results revealed significantly higher abstinence rates among those who adhered to the video-based computer tailoring condition across all educational groups. Additionally, in the text-based computer tailoring condition abstinence rates were significantly higher among smokers with a middle educational level who adhered to minimally one further session.

**Table 5:** Abstinence rates per educational level for the video-based and text-based computer tailoring interventions, stratified by adherence.

Condition				Abstinent n (%)	χ^2^_2_	*P* value
**Per educational level**						
	**Video condition (n=670)**				0.0	.99
		Low educational level		47 (20.9)		
		Middle educational level		51 (20.6)		
		High educational level		42 (21.2)		
	**Text condition (n=708)**				8.0	.02
		Low educational level		33 (14.3)		
		Middle educational level		41 (16.1)		
		High educational level		53 (23.9)		
**Stratified by adherence**						
	**Video condition (n=670)**					
		**Low educational level**			10.4	.001
			Adherence=0 (n=128)	17 (13.3)		
			Adherence>1 (n=97)	30 (30.9)		
		**Middle educational level**			16.5	<.001
			Adherence=0 (n=135)	15 (11.1)		
			Adherence>1 (n=112)	36 (32.1)		
		**High educational level**			29.9	.04
			Adherence=0 (n=113)	18 (15.9)		
			Adherence>1 (n=85)	24 (28.2)		
	**Text condition (n=708)**					
		**Low educational level**			3.5	.06
			Adherence=0 (n=218)	8 (8.9)		
			Adherence>1 (n=238)	25 (17.7)		
		**Middle educational level**			10.1	.001
			Adherence=0 (n=242)	8 (7.5)		
			Adherence>1 (n=260)	33 (22.3)		
		**High educational level**			3.4	.07
			Adherence=0 (n=177)	10 (15.6)		
			Adherence>1 (n=243)	43 (27.2)		

### Differences in Appreciation of the Program

Table 6 shows the program evaluation of sample 2. To test possible interaction effects between the conditions (video-based vs text-based computer tailoring) and educational level on process evaluation items, ANOVAs were conducted. There was no significant interaction between condition and educational level with respect to attention (*F*_1,327_=0.17, *P*=.84), comprehension (*F*_1,327_=1.59, *P*=.21), adaptation (*F*_1,327_=1.24, *P*=.29), appreciation (*F*_1,326_=0.07, *P*=.93), or processing (*F*_1,318_=0.008, *P*=.99) of the feedback messages. As shown in Table 6, the video feedback messages seem to be slightly better evaluated than the text-based messages in terms of appreciation and processing, but the differences did not reach significance.

**Table 6:** Means and standard deviations (SD) for evaluation of different aspects of the video-based and text-based computer tailoring intervention programs at 6-month follow-up.

Evaluation items	Overall sample (N=333)	Video (n=142)	Text (n=191)	*P* value
Feedback was attractive (attendance), mean (SD)	3.40 (1.04)	3.49 (1.07)	3.37 (1.03)	.18
Feedback was understandable (comprehensibility), mean (SD)	3.63 (0.70)	3.69 (0.70)	3.58 (0.70)	.15
Feedback fit to own situation (adaptation), mean (SD)	3.31 (0.74)	3.35 (0.78)	3.28 (0.71)	.40
Feedback was useful (appreciation), mean (SD)	3.54 (0.96)	3.64 (1.02)	3.45 (0.90)	.07
Feedback helped to make quit attempt (processing), mean (SD)	3.27 (0.86)	3.37 (0.90)	3.20 (0.82)	.06
Overall grade of feedback (from 1-10), mean (SD)	6.45 (1.62)	6.60 (1.67)	6.34 (1.57)	.15

## Discussion

### Principal Findings

The aim of this study was to evaluate the effects and appreciation of 2 multiple computer-tailored smoking cessation interventions (video- vs text-based messages) delivered via the Internet, regarding 6-month smoking abstinence among different educational groups. To our knowledge, this study is one of the first studies to test the effects of mode of delivery in the context of smoking cessation. Low levels of adherence may lead to an underestimation of the effects; therefore, the effectiveness of the 2 computer-tailored interventions was assessed by analyzing 2 samples. The first sample included all randomly assigned respondents who filled in the baseline questionnaire and followed the first session of the intervention whereas the second sample was a subsample of sample 1 including only respondents (in the experimental conditions) who adhered at least to one further session of the intervention.

Our study revealed several important findings. In contrast to our expectations, the results of all analyses revealed no significant differences in quit rates between smokers with low and high educational levels in the 2 experimental conditions (video- vs text-based messages). However, in both samples, the video-based computer-tailored smoking cessation intervention was effective in increasing 7-day point prevalence abstinence. The text-based computer-tailored smoking cessation intervention, however, was only significantly effective in increasing 7-day point prevalence abstinence in people who adhered to at least one further session (after baseline and session 1). The video-based condition was also more effective compared to the text-based condition regarding 7-day point prevalence abstinence in sample 2.

Moreover, with regard to prolonged abstinence our study revealed a differential effect of the intervention between people with a low or high readiness to quit, consistent with our second expectation. In sample 1, the video-based computer-tailored intervention appeared to be especially successful in increasing prolonged abstinence rates among smokers with a lower readiness to quit (within 4-6 months), whereas in sample 2, the video-based computer-tailored intervention was also effective among smokers willing to quit within 1 month. The multiple imputation and the complete case analyses yielded comparable results, a finding that may be attributed to the fact that rates of missing data were not extremely high at 6-month follow-up (on average 30%).

Consistent with previous findings [33,34] in other behavioral domains, the results of our study indicate that tailored video-based messages might be more effective in supporting smokers to quit smoking regarding 7-day point prevalence abstinence compared to tailored text-based messages. Using video-based messages was equally effective in smokers of all educational levels. This is in-line with past research which found that Internet users appreciate the concept of tailored video interventions over text-based interventions [27]. Contrary to our first hypothesis, the video-based computer-tailored intervention was not more effective in less-educated groups than in highly educated groups. The delivery of information on the Internet is rapidly changing with the proportion of video content increasing [28]; therefore, it is possible that there is a general preference for receiving tailored information in a video format. In-line with this, our program evaluation also revealed a slightly better evaluation of the tailored video messages. Again, no differences were recognized between lower- and higher-educated respondents.

Our study revealed another interesting effect of the video-tailored intervention for people with a lower readiness to quit smoking. With different routings available in our smoking cessation intervention, we expected that both interventions would be effective for people with a lower motivation to quit at baseline. Partially consistent with our second hypothesis, the results revealed that only the video-tailored intervention appeared to be successful in smokers with a lower readiness to quit. The availability of different intervention routings provided these less-motivated smokers the possibility to reflect on their smoking behavior and their potential quit attempt; these less-motivated smokers may have benefited from this option in the video-based condition.

Consistent with our expectation, our study showed that abstinence rates were higher overall when respondents adhered to at least one further intervention element. In sample 2, adherence can be regarded as a determinant of the efficacy of the program. These findings are in-line with different previous research, which also found that the efficacy of a program increased when people adhered to the intervention [7,13].

### Strengths and Limitations

To our knowledge, this is the first study to assess the effectiveness of a Web-based tailored video and text intervention aiming to promote smoking cessation in groups with varying levels of education and varying levels of readiness to quit. A strength of the study is that 2 different sensitivity analyses were performed to test the robustness of our results. However, our study is also subject to several limitations. First, a misreport may have occurred when respondents were asked to indicate their smoking status at the 6-month follow-up measurement. For financial reasons, we were not able to biochemically validate respondents’ self-assessed smoking status. Although future Web-based intervention studies might be recommended to verify smoking status through the use of biochemical cotinine test as part of a more detailed follow-up assessment, it is also argued that this might be irrelevant (eg, if anonymity has been guaranteed) [55]. It may be that Web-based interventions are attractive because they enable people to participate anonymously. This topic requires further elaboration in future studies. Second, we assessed smoking status after 6 months; however, it might be valuable to replicate these findings and investigate whether these results persist over a longer follow-up period. Lastly, during our intervention, respondents were not able to choose a quit date within a week of baseline. Although there is value in taking advantage of current motivational readiness and not delaying an attempt, this could be seen as a weakness of our intervention [56].

Despite these limitations, the present study provides evidence that video-based messages are successful in stimulating quitting behavior. As past research has already indicated that Internet users prefer to receive content in the form of video-based messages [27] and our results confirmed this, the use of audiovisual content might increase the appeal of future health interventions and smoking cessation interventions in particular.

### Conclusions

The current study provides important new evidence for the effectiveness of a video-based computer-tailored smoking cessation intervention. The results suggest that a video-based computer-tailored intervention with personalized feedback adapted to the smokers’ motivation to quit might be effective in increasing abstinence rates for smokers with different educational levels. The results support the feasibility of using video messaging to affect smoking behavior. We measured smoking abstinence after 6 months; more research is needed to examine whether these results persist over longer follow-up periods.

## Multimedia Appendix 1

Results of regression analysis on sample 2.

## Multimedia Appendix 2

Results of complete case regression analysis.

## Multimedia Appendix 3

CONSORT-EHEALTH checklist V1.6.2 [57].

## Abbreviations

CES-D: Center for Epidemiologic Studies Depression scale
COPD: chronic obstructive pulmonary disease
FTND: Fagerström Test for Nicotine Dependence
GP: general practitioner
HSD: honestly significant difference
MREC: Medical Research Ethics Committee
