# Supplementary material for: Comparison of Text and Video Computer-Tailored Interventions for Smoking Cessation: Randomized Controlled Trial
Source: J Med Internet Res. 2014 Mar 3;16(3):e69. doi: 10.2196/jmir.3016 (PMC3961744; doi:10.2196/jmir.3016)
Supplement: Supplementary file 1 [file jmir_v16i3e69_app1.pdf]

Multimedia Appendix 1. Results of regression analysis on sample 2

Table 1 [Six-month abstinence rates (Seven-day point prevalence abstinence and prolonged abstinence) per condition for sample 2 (negative scenario)]

Negative scenario

	Total(N)	Video	Text	Control	p-value
Seven-day PPA [a] % (N)	1462	30.6% (90)	22.6% (101)	14.6% (105)	**.000**
PA [b] % (N)	1462	22.8% (67)	33.3% (77)	37.7% (87)	**.000**
PA [b] % (N)	1462				
Readiness to quit within 1 month		25.1% (45)	21.4% (60)	15.2% (52)	**.02**
Readiness to quit within 1-3 months		17.1% (13)	10.6% (11)	13.2% (30)	.44
Readiness to quit within 4-6 months		23.1% (9)	9.5% (6)	3.3% (5)	**.000**

[a] Point prevalence abstinence (PPA) ; [b] Prolonged abstinence (PA)

Table 2 [Factors associated to seven-day point prevalence abstinence in sample 2 (negative scenario) in the present study]

Negative scenario	Sample 2 (N=1462)		
Variable	OR	95% CI	p-value
VCT vs. CC	2.29	1.64-3.20	**.000**
TCT vs. CC	1.57	1.15-2.15	**.005**
Gender (male)	.88	.66-1.16	.35
Age	1.01	.99-1.02	.19
Dutch nationality	1.24	.65-2.39	.51
Middle education level [a]	1.13	.81-1.57	.46
High education level [a]	1.07	.75-1.52	.70
Readiness to quit within 1 month [b]	1.64	1.06-2.55	**.02**
Readiness to quit within 1-3 months [b]	1.40	.88-2.22	.16
FTND score	.94	.89-1.00	.05
CES-D score	.94	.89-1.00	.07
With COPD [c]	.93	.60-1.43	.74
With cancer [c]	.89	.31-2.5	.82
With diabetes [c]	.98	.53-1.84	.96
With cardiovascular diseases [c]	1.17	.74-1.86	.49
With asthma [c]	1.21	.69-2.14	.50
Recruitment strategy Newspaper/Internet [d]	.63	.41-.98	**.04**
Preparatory planning	1.09	1.03-1.15	**.005**
Coping planning	.99	.91-1.08	.87
Self-efficacy	1.15	.98-1.35	.08

Note: p-values <.05 are marked bold; interaction terms are not included in the final model since they were not significant and ORs are adjusted for variables significant at baseline and drop-out; [a] low education is the reference category; [b] willingness to quit within 4-6 months is the reference category;
[c] not suffering from the disease is the reference category; [d] general practitioner (GP) is the reference category

Table 3 [Factors associated to prolonged abstinence in sample 2 (negative scenario) in the present study]

Negative scenario	Sample 2 (N=1462)		
Variable	OR	95% CI	*p*-value
VCT vs. CC	8.99	2.75-29.41	**.000**
TCT vs. CC	3.13	.91-10.77	.07
Gender (male)	.77	.56-1.05	.10
Age	1.02	1.00-1.03	**.030**
Dutch nationality	1.12	.52-2.40	.77
Middle Education level [a]	1.08	.75-1.55	.69
High education level [a]	.91	.62-1.34	.63
Readiness to quit within 1 month [b]	4.14	1.59-10.79	**.004**
Readiness to quit within 1-3 months [b]	4.10	1.59-10.79	**.005**
FTND score	.95	.89-1.01	.09
CES-D score	.91	.85-.98	**.01**

With COPD [c]	1.20	.74-1.95	.46
With cancer [c]	.69	.24-1.97	.49
With diabetes [c]	.90	.46-1.77	.77
With cardiovascular diseases [c]	1.34	.80-2.24	.27
With asthma	1.22	.65-2.30	.54
Recruitment strategy	.63	.39-1.01	**.05**
Newspaper/Internet [d]			
Preparatory planning	1.12	1.05-1.20	**.001**
Coping planning	1.06	.96-1.18	.24
Self-efficacy	1.16	.98-1.39	.09
**Interactions**			
High readiness to quit * VCT	.18	.05-.66	**.009**
High readiness to quit * TCT	.47	.13-1.73	.25
Middle readiness to quit * VCT	.13	.03-.53	**.004**
Middle readiness to quit * TCT	.24	.05-.99	**.05**
**Subgroup analyses**			
**Readiness to quit within 1 month**			
VCT vs. TCT	1.13	.71-1.79	.60
VCT vs. CC	1.65	1.04-2.63	**.03**
TCT vs. CC	1.46	.95-2.24	.08
**Readiness to quit within 1-3 months**			
VCT vs. TCT	1.61	.66-3.91	.29
VCT vs. CC	1.18	.57-2.46	.65
TCT vs. CC	.74	.35-1.56	.42
**Readiness to quit 4-6 within months**			
VCT vs. TCT	2.88	.91-9.06	.07
VCT vs. CC	8.99	2.75-29.41	**.000**
TCT vs. CC	3.13	.91-10.77	.07

Note: p-values <.05 are marked bold and ORs are adjusted for variables significant at baseline and drop-out; [a] low education is the reference category; [b] willingness to quit within 4-6 months is the reference category; [c] suffering not from the disease is the reference category; [d] general practitioner (GP) is the reference category
