# Supplementary material for: Comparison of Text and Video Computer-Tailored Interventions for Smoking Cessation: Randomized Controlled Trial
Source: J Med Internet Res. 2014 Mar 3;16(3):e69. doi: 10.2196/jmir.3016 (PMC3961744; doi:10.2196/jmir.3016)
Supplement: Supplementary file 2 [file jmir_v16i3e69_app2.pdf]

Multimedia Appendix 2. Results of complete case regression analyses.

Table 1 [Factors associated to seven-day point prevalence abstinence in samples 1 and 2 (complete case analysis) in the present study]

CC analysis	Sample 1 (N=2099)			Sample 2 (N=1462)		
Variable	OR	95% CI	*p*-value	OR	95% CI	*p*-value
VC vs. CC	1.76	1.30-3.40	**.000**	2.40	1.68-3.44	**.000**
TC vs. CC	1.30	.96-1.77	.09	1.49	1.07-2.07	**.018**
Gender (male)	.88	.68-1.14	.32	.88	.65-1.18	.39
Age	1.00	.99-1.01	.45	1.00	.99-1.02	.60
Dutch nationality	1.75	.94-3.27	.08	1.68	.81-3.45	.16
Middle education level [a]	1.15	.85-1.55	.37	1.23	.87-1.75	.23
High education level [a]	1.20	.87-1.65	.27	1.18	.81-1.70	.39
Readiness to quit within 1 month [b]	1.96	1.32-2.92	**.001**	2.02	1.28-3.19	**.002**
Readiness to quit within 1-3 months [b]	1.54	1.02-2.33	**.04**	1.54	.95-2.48	.08
FTND score	.95	.90-1.00	**.04**	.94	.88-.99	**.03**
CES-D score	.94	.89-1.00	**.04**	.94	.88-1.01	.07
With COPD [c]	1.11	.75-1.64	.60	.95	.60-1.49	.82
With cancer [c]	.91	.34-2.43	.85	.92	.31-2.77	.89
With diabetes [c]	1.14	.61-2.14	.68	.93	.48-1.79	.83
With cardiovascular diseases [c]	1.11	.71-1.36	.65	1.16	.72-1.88	.54
With asthma	.85	.53-1.37	.50	1.17	.64-2.14	.61
Recruitment strategy Newspaper/Internet [d]	.59	.38-.90	**.02**	.54	.35-.93	**.03**
Preparatory planning	1.08	1.02-1.13	**.007**	1.09	1.03-1.16	**.005**
Coping planning	1.01	.92-1.10	.92	.98	.88-1.08	.67
Self-efficacy	1.11	.95-1.29	.18	1.14	.96-1.34	.13

Note: p-values <.05 are marked bold, ORs are adjusted for variables significant at baseline and drop-out and interaction terms are not included in the final model since they were not significant; [a] low education is the reference category; [b] willingness to quit within 4-6 months is the reference category; [c] suffering not from the disease is the reference category; [d] general practitioner (GP) is the reference category

Table 2 [Factors associated to prolonged abstinence in samples 1 and 2 (complete case analysis) in the present study]

Complete case analysis	Sample 1 (N=2099)			Sample 2 (N=1462)		
Variable	OR	95% CI	*p*-value	OR	95% CI	*p*-value
VCT vs. CC	7.20	2.38-21.75	**.000**	8.79	2.60-29.75	**.000**
TCT vs. CC	2.80	.90-.8.66	**.006**	2.67	.76-9.36	.13
Gender (male)	.66	.49-.88	**.006**	.75	.53-1.05	.09
Age	1.01	.99-1.02	.23	1.02	1.00-1.03	.11
Dutch nationality	1.90	.93-3.84	.08	1.50	.64-3.47	.35
Middle education level [a]	1.27	.90-1.79	.18	1.19	.81-1.8	.38
High education level [a]	1.02	.71-1.48	.91	.98	.65-1.48	.92
Readiness to quit within 1 month [b]	5.46	2.06-14.45	**.001**	5.29	1.99-14.07	**.001**
Readiness to quit within 1-3 months [b]	4.49	1.65-12.16	**.003**	4.43	1.63-12.03	**.004**
FTND score	.94	.89-1.00	**.04**	.95	.88-1.01	.10
CES-D score	.90	.83-.96	**.002**	.90	.83-.97	**.009**
With COPD [c]	1.40	.89-2.20	.14	1.33	.80-2.22	.28
With cancer [c]	.55	.20-1.53	.25	.66	.21-2.04	.47
With diabetes [c]	1.07	.54-2.12	.84	.98	.48-1.99	.95
With cardiovascular diseases [c]	1.25	.76-2.06	.37	1.40	.81-2.41	.23
With asthma [c]	1.13	.65-1.97	.66	1.27	.65-2.50	.49
Recruitment strategy Newspaper/Internet [d]	.50	.31-.81	**.005**	.51	.30-.87	**.01**
Preparatory planning	1.08	1.02-1.15	**.009**	1.12	1.05-1.20	**.001**
Coping planning	1.07	.97-1.18	.18	1.07	.95-1.19	.25
Self-efficacy	1.09	.92-1.29	.33	1.10	.91-1.32	.33
**Interactions**						
High readiness to quit * VCT	.18	.05-.59	**.005**	.20	.05-.76	**.02**
High readiness to quit * TCT	.45	.13-1.52	.20	.53	.140-2.01	.35
Middle readiness to quit * VCT	.14	.04-.51	**.003**	.15	.04-.63	**.01**
Middle readiness to quit * TCT	.25	.07-.93	**.039**	.23	.05-1.00	**.05**
**Subgroup analyses**						
**Readiness to quit within 1 month**						
VCT vs. TCT	1.01	.66-1.54	.96	1.26	.76-2.08	.37
VCT vs. CC	1.28	.82-2.01	.28	1.77	1.07-2.95	**.03**
TCT vs. CC	1.27	.82-1.96	.29	1.41	.89-2.23	.15
**Readiness to quit within 1-3 months**						
VCT vs. TCT	1.45	.72-2.92	.30	2.16	.85-5.46	.11
VCT vs. CC	1.02	.54-1.92	.94	1.32	.61-2.88	.48

TCT vs. CC	.71	.38-1.36	.30	.61	.28-1.32	.21
**Readiness to quit within 4-6 months**						
VCT vs. TCT	2.57	1.03-6.44	**.04**	3.29	1.01-10.76	**.05**
VCT vs. CC	7.20	2.38-21.75	**.000**	8.79	2.60-29.75	**.000**
TCT vs. CC	2.80	.90-8.66	**.006**	2.67	.76-9.36	.13

Note: p-values <.05 are marked bold and ORs are adjusted for variables significant at baseline and drop-out and; [a] low education is the reference category; [b] willingness to quit within 4-6 months is the reference category; [c] suffering not from the disease is the reference category; [d] general practitioner (GP) is the reference category
