# Supplementary material for: Comparison of Text and Video Computer-Tailored Interventions for Smoking Cessation: Randomized Controlled Trial
Source: J Med Internet Res. 2014 Mar 3;16(3):e69. doi: 10.2196/jmir.3016 (PMC3961744; doi:10.2196/jmir.3016)
Supplement: Supplementary file 3 [file jmir_v16i3e69_app3.pdf]

CONSORT-EHEALTH Checklist V1.6.2 Report

(based on CONSORT-EHEALTH V1.6), available at [http://tinyurl.com/consort-ehealth-v1-6].

3016

Date completed

10/10/2013 9:30:15

by

Nicola Esther Stanczyk

Effects of two web-based computer-tailored interventions for smoking cessation: Comparing text and video delivery of messages.

TITLE

1a-i) Identify the mode of delivery in the title

YES:
Effects of two web-based computer-tailored interventions for smoking cessation: Comparing text and video delivery of messages

1a-ii) Non-web-based components or important co-interventions in title

YES:
Effects of two web-based computer-tailored interventions for smoking cessation: Comparing text and video delivery of messages

1a-iii) Primary condition or target group in the title

n/a

ABSTRACT

1b-i) Key features/functionalities/components of the intervention and comparator in the METHODS section of the ABSTRACT

Objective: The results of video computer tailoring (VCT) and text computer tailoring (TCT) were compared with the results of a control condition (CC). Main effects and differential effects for subgroups with different educational levels and different levels of readiness to quit were assessed.

1b-ii) Level of human involvement in the METHODS section of the ABSTRACT

During a blind randomized controlled trial,
smokers willing to quit within 6 months were assigned to VCT (N=670), TCT (N=708) or CC (short generic text advice) (N=721).
After 6 months, effects on 7-day point prevalence abstinence (PPA) and prolonged abstinence (PA) were assessed using logistic regression analyses. Analyses were conducted in two samples:

1b-iii) Open vs. closed, web-based (self-assessment) vs. face-to-face assessments in the METHODS section of the ABSTRACT

self-assessment

1b-iv) RESULTS section in abstract must contain use data

VCT was more effective in increasing 7-day PPA than CC in both samples (OR= 1.45, p= .010; OR= 2.29, p= .000).
TCT was only more effective than CC in increasing 7-day PPA in sample 2.
VCT resulted in significantly higher PA rates than CC among smokers with high (ready to quit within 1 month) and low (ready to quit within 4-6 months) readiness to quit, in sample 2
(OR= 4.49 p= .008; OR= 9.35, p= .001). No differential effects were found for level of education.
Complete case analyses and multiple imputation yielded similar results.

1b-v) CONCLUSIONS/DISCUSSION in abstract for negative trials

Results suggest that video-based messages with personalized feedback adapted to the smoker's motivation to quit might be effective in increasing abstinence rates for smokers with diverse educational levels.

INTRODUCTION

2a-i) Problem and the type of system/solution

A wide range of different smoking cessation interventions have been developed and implemented but in spite of this smoking rates remain high, especially among people with a lower level of education [1-4]. This illustrates the need to improve smoking cessation intervention strategies for this group.

2a-ii) Scientific background, rationale: What is known about the (type of) system

Although web-based CT smoking cessation interventions have been shown to be potentially effective [15, 16], they often report problems in attracting, engaging and retaining smokers and quitters [8, 17-20].

METHODS

3a) CONSORT: Description of trial design (such as parallel, factorial) including allocation ratio

We expected video computer tailoring (VCT) to be more effective in smokers with a lower level of education, whereas text computer tailoring (TCT) was expected to be more effective in smokers with a higher level of education.
As the interventions included different routings tailored according to the smokers' readiness to quit, we furthermore expected less motivated smokers to be successful in their quit attempt.

3b) CONSORT: Important changes to methods after trial commencement (such as eligibility criteria), with reasons

n/a

3b-i) Bug fixes, Downtimes, Content Changes

n/a

4a) CONSORT: Eligibility criteria for participants
Respondents were eligible for participation when they
were motivated to quit smoking within the following 6 months,
when they were 18 years or older and when they had
access to the Internet.
4a-i) Computer / Internet literacy
n/a
4a-ii) Open vs. closed, web-based vs. face-to-face assessments:
Of the 2551 potential respondents assessed for eligibility,
overall 49 declined to participate, 138 did not meet inclusion criteria
and 265 did not complete the baseline questionnaire or had no
baseline quit date (within routing 1). Finally, 2106 respondents
were randomized to the
VCT condition (N=670), TCT condition (N=708) and the CC (N=721).
4a-iii) Information giving during recruitment
First, a random sample of about 150 general practitioners (GPs)
was asked to refer smoking patients to the intervention website.
GP practices were provided with recruitment materials (flyers,
business cards etc.) for this purpose.
Second, respondents were also recruited to participate via advertising
campaigns in local newspapers, newspaper websites and health fund
websites. At last, we used several national and international online
social networking websites such as Hyves and Facebook to invite smokers to participate in our smoking cessation study. All advertisements provided a link to the intervention website that enabled
people to find more information about the intervention and participation.
4b) CONSORT: Settings and locations where the data were collected
n/a
4b-i) Report if outcomes were (self-)assessed through online questionnaires
self-assessed
4b-ii) Report how institutional affiliations are displayed

5) CONSORT: Describe the interventions for each group with sufficient details to allow replication, including how and when they were actually administered
5-i) Mention names, credential, affiliations of the developers, sponsors, and owners
This work was supported by ZonMw, the Netherlands
Organisation for Health Research and Development
(grant number: 20011007).
5-ii) Describe the history/development process
n/a
5-iii) Revisions and updating
no
5-iv) Quality assurance methods

5-v) Ensure replicability by publishing the source code, and/or providing screenshots/screen-capture video, and/or providing flowcharts of the algorithms used

5-vi) Digital preservation
www.steunbijstoppen.nl
5-vii) Access
Participants did not had to pay for the intervention.
5-viii) Mode of delivery, features/functionalities/components of the intervention and comparator, and the theoretical framework
video versus text messages
The content of the feedback messages was exactly the same in both
the text and video condition. In the text condition, respondents
received multiple sessions of CT text-based advice
without any graphics or animations. In the video condition,
the same tailored advice were presented by adults in a video message.
Five different adult presenters (two males, three females) were selected
out of a screening test of 20 persons and delivered the tailored advice
in a TV ‘news program’ format. We used a mix of adults during the different
sessions who presented the different tailored advices.
5-ix) Describe use parameters

5-x) Clarify the level of human involvement
The MREC decided that no MREC approval was necessary because
respondents were not required to undertake any particular action.
5-xi) Report any prompts/reminders used

Respondents in the text and video condition received tailored feedback
over three months (see Intervention and Figure.1 for details).
After 6 months follow-up, all respondents were sent an email invitation with a link to the intervention website to fill out the 6-month follow-up measurement. Respondents who did not complete the follow-up measurement after one week were reminded by email to fill out the online questionnaire. A further reminder was sent after two weeks if necessary. Respondents who did not respond to the email invitation or the two reminders received another email, inviting them to briefly indicate their current smoking status. This email requested completion of a shortened version of the 6-month follow-up measurement, consisting of 10 (instead of 95) important smoking related questions, which they could return by email. Lastly, if this abbreviated email assessment was still not completed, respondents were called for a short telephone interview, asking the same questions as in the shortened online questionnaire.

5-xii) Describe any co-interventions (incl. training/support)

n/a

6a) CONSORT: Completely defined pre-specified primary and secondary outcome measures, including how and when they were assessed

At the 6-month follow-up measurement,
7-day point prevalence abstinence (PPA)
was self-assessed by one item asking respondents whether they had refrained from smoking during the last seven days (0=no; 1= yes) [48, 49].
In addition, prolonged abstinence (PA) was self-assessed by one item asking respondents whether they had refrained from smoking since their last quit attempt (allowing for a two-week grace period during which the respondent could smoke 1-5 cigarettes) (0=no;1=yes) [48, 49].
In line with the definition of PA, those who reported that they had quit less than three months before the follow-up measurements were not included as quitters in the PA measurement [49].

6a-i) Online questionnaires: describe if they were validated for online use and apply CHERRIES items to describe how the questionnaires were designed/deployed

see study protocol

6a-ii) Describe whether and how “use” (including intensity of use/dosage) was defined/measured/monitored

See Figure 1 in paper

6a-iii) Describe whether, how, and when qualitative feedback from participants was obtained

n/a

6b) CONSORT: Any changes to trial outcomes after the trial commenced, with reasons

no

7a) CONSORT: How sample size was determined

7a-i) Describe whether and how expected attrition was taken into account when calculating the sample size

7b) CONSORT: When applicable, explanation of any interim analyses and stopping guidelines

n/a

8a) CONSORT: Method used to generate the random allocation sequence

n/a

8b) CONSORT: Type of randomisation; details of any restriction (such as blocking and block size)

n/a

9) CONSORT: Mechanism used to implement the random allocation sequence (such as sequentially numbered containers), describing any steps taken to conceal the sequence until interventions were assigned

n/a

10) CONSORT: Who generated the random allocation sequence, who enrolled participants, and who assigned participants to interventions

n/a

11a) CONSORT: Blinding - If done, who was blinded after assignment to interventions (for example, participants, care providers, those assessing outcomes) and how

11a-i) Specify who was blinded, and who wasn’t

They did not know to which study group they were allocated.

11a-ii) Discuss e.g., whether participants knew which intervention was the “intervention of interest” and which one was the “comparator”

n/a

11b) CONSORT: If relevant, description of the similarity of interventions

n/a

12a) CONSORT: Statistical methods used to compare groups for primary and secondary outcomes

Third, logistic regression analyses were conducted to investigate the effectiveness of the intervention on the outcome measures assessed at the 6-month follow-up measurements.
The analyses were performed adjusting for potential confounders, including demographic variables (e.g. age, educational level, gender and ethnicity) and variables with a theoretically expected disturbing effect (e.g. addiction level, recruitment strategy, readiness to quit smoking, depression, smoking related illnesses, self-efficacy, preparatory planning and coping planning), baseline differences, drop-out predictors and two interaction terms (readiness to quit smoking by condition and educational level by condition). Where significant interaction terms were found, stratified analyses were performed separately for each group.

12a-i) Imputation techniques to deal with attrition / missing values

Intention to treat: negative scenario & multiple imputation

12b) CONSORT: Methods for additional analyses, such as subgroup analyses and adjusted analyses

Where significant interaction terms were found, stratified analyses were performed separately for each group.

RESULTS

13a) CONSORT: For each group, the numbers of participants who were randomly assigned, received intended treatment, and were analysed for the primary outcome

We added an attrition diagram in which these results are shown.

13b) CONSORT: For each group, losses and exclusions after randomisation, together with reasons

We added an attrition diagram in which these results are shown.

13b-i) Attrition diagram

14a) CONSORT: Dates defining the periods of recruitment and follow-up

n/a

14a-i) Indicate if critical "secular events" fell into the study period

14b) CONSORT: Why the trial ended or was stopped (early)

n/a

15) CONSORT: A table showing baseline demographic and clinical characteristics for each group

See our flow chart

15-i) Report demographics associated with digital divide issues

see table 1

16a) CONSORT: For each group, number of participants (denominator) included in each analysis and whether the analysis was by original assigned groups

16-i) Report multiple "denominators" and provide definitions

See table 3 & 4

16-ii) Primary analysis should be intent-to-treat

17a) CONSORT: For each primary and secondary outcome, results for each group, and the estimated effect size and its precision (such as 95% confidence interval)

See Table in paper

17a-i) Presentation of process outcomes such as metrics of use and intensity of use

17b) CONSORT: For binary outcomes, presentation of both absolute and relative effect sizes is recommended

See table 2 & 3 & 4

18) CONSORT: Results of any other analyses performed, including subgroup analyses and adjusted analyses, distinguishing pre-specified from exploratory

We also compared the effects of the intervention between low and high motivated smokers.

18-i) Subgroup analysis of comparing only users

19) CONSORT: All important harms or unintended effects in each group

No intended harms were reported.

19-i) Include privacy breaches, technical problems

19-ii) Include qualitative feedback from participants or observations from staff/researchers

DISCUSSION

20) CONSORT: Trial limitations, addressing sources of potential bias, imprecision, multiplicity of analyses

20-i) Typical limitations in ehealth trials

First, some degree of recall bias may have occurred when respondents were asked to indicate their smoking status at the 6-month follow-up measurement. For financial reasons, we were unfortunately not able make a biochemical validation of participants' self-assessed smoking status. Although future web-based intervention studies might be recommended to verify smoking status through the use of biochemical cotinine test as part of a more detailed follow-up assessment, it is also argued that this might be irrelevant, e.g. if anonymity has been guaranteed [54]. It may be that web-based interventions are attractive because they enable people to participate anonymously. This topic requires further elaboration in future studies. Second, we assessed smoking status after 6 months; however it might be valuable to replicate these findings and investigate whether these results persist over a longer follow-up period.

21) CONSORT: Generalisability (external validity, applicability) of the trial findings

21-i) Generalizability to other populations

21-ii) Discuss if there were elements in the RCT that would be different in a routine application setting

22) CONSORT: Interpretation consistent with results, balancing benefits and harms, and considering other relevant evidence

22-i) Restate study questions and summarize the answers suggested by the data, starting with primary outcomes and process outcomes (use)

Our study revealed several important findings. First, in both samples, the web-based video-based multiple CT smoking cessation intervention was effective in increasing 7-day PPA. The text-based multiple CT smoking cessation intervention however was only significantly effective in increasing 7-day PPA in people who adhered to minimal one session. The VCT condition was also more effective compared to the TCT condition, regarding 7-day PPA in people who adhered to minimal one session. Secondly, with regard to PA our study revealed only a differential effect of the intervention for people with a low or high readiness to quit.

22-ii) Highlight unanswered new questions, suggest future research

Other information

23) CONSORT: Registration number and name of trial registry

Netherlands Trial Register (NTR3102).

24) CONSORT: Where the full trial protocol can be accessed, if available

n/a

25) CONSORT: Sources of funding and other support (such as supply of drugs), role of funders

This work was supported by ZonMw, the Netherlands Organisation for Health Research and Development (grant number: 20011007).

X26-i) Comment on ethics committee approval

The current study was submitted for approval to the Medical Research Ethics Committee (MREC) of Atrium Medical Centre Heerlen. The MREC decided that no MREC approval was necessary because respondents were not required to undertake any particular action. The study was registered at the Dutch Trial Register (NTR3102).
The study was in line with the ethical codes of conduct of the APA [38].

x26-ii) Outline informed consent procedures

Online informed consent

X26-iii) Safety and security procedures

X27-i) State the relation of the study team towards the system being evaluated

Hein de Vries is scientific director of Vision2-Health, a company that licences evidence-based computer-tailored health communication tools.
